# Pivot shift intraoperative quantitative assessment using a smartphone accelerometer in ACL deficient knees

**DOI:** 10.1186/s40634-023-00570-7

**Published:** 2023-01-25

**Authors:** Guillaume Soudé, Jean-Baptiste De Villeneuve Bargemon, Raghbir Khakha, Martine Pithioux, Jean-Noël Argenson, Matthieu Ollivier, Christophe Jacquet

**Affiliations:** 1 Aix-Marseille University, CNRS, ISM UMR 7287, 13288, cedex 09 Marseille, France; 2 Department of Orthopedic surgery and Traumatology St. Marguerite Hospital, Institute of movement and locomotion, 270 Boulevard Sainte Marguerite, 29 13274 Marseille, BP France; 3 International Wrist Center, Bizet Clinic, Paris, France; 4 Guys and St Thomas Hospitals, London, UK

**Keywords:** PS test, Anterior cruciate ligament repair, Triaxial smartphone accelerometer, Knee laxity, Meniscus injury, Protocol study

## Abstract

**Purpose:**

The Pivot Shift (PS) test is a complex clinical sign that assesses the internal rotation and anterior tibial translation, which occurs abnormally in ACL deficient-knees. Because of the high inter-observer variability, different devices have been designed to characterize this complex movement in quantitative variables. The objective of this pilot study is to validate the reproducibility of intraoperative quantitative assessment of the PS with a smartphone accelerometer.

**Methods:**

Twelve ACL-injured knees were included and compared with the contralateral uninjured side. The PS was measured by two independent observers utilizing a smartphone accelerometer and graded according to the IKDC classification. Measurements were taken preoperatively, intraoperatively and postoperatively. Intraoperative readings were taken during each stage of reconstruction or repair of meniscoligamentous lesions including meniscal lesions, ramp lesions, ACL reconstruction and lateral tenodesis. Reproducibility of the measurements were evaluated according to an intraclass correlation coefficient (ICC).

**Results:**

The intra-observer reliability was good for the first examiner and excellent for the second examiner, with the ICC 0.89 [0.67, 0.98] *p* < 0,001 and ICC 0.97 [0.91, 1.0] *p* < 0,001 respectively. The inter-observer reliability was excellent between the two observers with the ICC 0.99 [0.97, 1.0] *p* < 0,001. The mean tibial acceleration measured 3.45 m.s^2^ (SD = 1.71) preoperatively on the injured knees and 1.03 m.s^2^ (SD = 0.36) on the healthy knees, demonstrating a significant difference following univariate analysis *p* < 0.001. Postoperatively, no significant difference was observed between healthy and reconstructed knees The magnitudes of tibial acceleration values were correlated with the PS IKDC grade.

**Conclusion:**

The smartphone accelerometer is a reproducible device to quantitatively assess the internal rotation and anterior tibial translation during ACL reconstruction surgery. The measurements are influenced by the different surgical steps. Other larger cohort studies are needed to evaluate the specific impact of each step of the ACL reconstruction and meniscal repair on this measurement. An external validation using other technologies are needed to validate the reliability of this device to assess the PS test.

**Level of evidence:**

Level IV, case series, pilot study.

## Introduction

The Pivot Shift (PS) test is a complex clinical sign that assesses the internal rotation and anterior tibial translation, which occurs abnormally in ACL deficient-knees [5]. In the presence of an ACL injury, the PS test has a specificity ranging from 0 to 97 to 0–99 and sensitivity ranging from 0 to 18 to 0–48 [23]. The pathological nature of this test is based on a subjective evaluation in 3 grades: grade 1 = glide guard, 2: clunk and grade 3: gross instability [9]. The exact pathophysiology of the PS remains poorly understood and incompletely accepted [30]. A multifactorial background involving multiple stabilizing anatomical structures of the knee seems to be the most likely hypothesis [17]. The PS test is correlated to several factors including the anterolateral complex [26], meniscal lesions (posterolateral and posteromedial [16] specifically), iliotibial band or Kaplan Fibers [3] and the bone morphology of the tibial plateau [25]. Because of the high inter-observer variability, different devices have been designed to characterize this complex movement in quantitative variables [12]. Multiple studies have investigated the use of accelerometers [14, 22]. Accelerometers using smartphone technologies have the advantages of being easy to use and reliable [2, 7, 22]. However, accelerometers have never been used intra-operatively as part of clinical practice.

The main objective of this pilot study is to validate the reproducibility of intraoperative quantitative assessment of the PS test with a smartphone accelerometer. The secondary objective was to identify potential parameters influencing the PS during ACL reconstruction surgery.

The hypothesis of this work was that the use of a smartphone accelerometer intra-operatively is a reproducible and reliable method to measure the PS, and the measurements could be influenced by the different surgical steps.

## Methods

All patients provided written consent, and the study was approved by our hospital’s ethics committee. (CPP N°2020-A00910033). A prospective analysis was performed on 12 patients who underwent ACL reconstructions between May 2021 and February 2022.

The inclusion criteria were defined as patients aged between 16 and 45 years old undergoing primary ACLR without associated collateral ligament (≤ MRI grade 2) surgery. All patients had clinical anterior instability and complete ACL tear on MRI.

Exclusion criteria were defined as history of ipsilateral knee surgery, an associated bone or cartilage procedure, tibial spine avulsion, pathological hyperlaxity (Beighton score > 3), previous history of contralateral ACLR, multi-ligament injury confirmed by MRI, chronic inflammatory joint disease.

All patients provided written consent, and the study was approved by our hospital’s ethics committee. (CPP N°2020-A00910033).

### Experimental protocol

All patients were operated by two senior surgeons using two different ACLR techniques with different types of grafts and a Lateral Extra-articular Tenodesis (LET). The choice of surgical techniques was based on individual surgeons’ preferences.

### Surgical ACLR technique

- Quadriceps tendon + LET using the modified Lemaire technique (QT + LET group) [10]
- Combined ACL and Anterolateral Ligament Reconstruction (DT3I2 group) [24]

### Meniscal repair techniques

- For posterior and middle segments, all-inside meniscal repair techniques were used (AIR +®, Stryker, Mahwah NJ, USA).
- For the anterior segment, an outside-in suture technique was used:
- For ramp lesions, a single posteromedial approach was used with a 25 curved suture hook device (SutureLasso; Arthrex, Naples, FL) loaded with a No. 1 absorbable monofilament suture (PDS; Ethicon, Somerville, NJ) [28].

### Data collection

Two senior orthopedic surgeons were blinded to the accurate diagnosis for the pre-operative measurements (ie information regarding meniscal status was withheld). They independently performed the PS test on the patient under anesthesia, firstly on the healthy limb and then on the limb to be operated on. All pre-operative measurements were repeated three times by each examiner. For a right knee, the examiner placed themselves on the right side of the patient who was in the supine position. The left hand then held the foot and elevated it from the plane of the bed (about 20° of hip flexion and abduction). The limb was placed in slight internal rotation, while the right hand positioned the knee in slight flexion (20°), placed in the proximal third of the leg, applying a valgus stress. Large forces and extreme rotations were avoided. The dynamic phase of the test consisted of gradually flexing the knee in a passive manner. The abrupt subluxation then reduction of the lateral tibial plateau under the femoral condyles occurred between 30° and 60° of flexion. This reduction was described according to the international IKDC classification in 3 stages, grade 1 = glide guard 2: clunk, and grade 3: gross instability, and the assessment was made according to the examiner’s perception of the contralateral healthy side [19]. The technique was standardized in order to be reproducible and to limit the variability of the results [8].

**Fig. 1:**
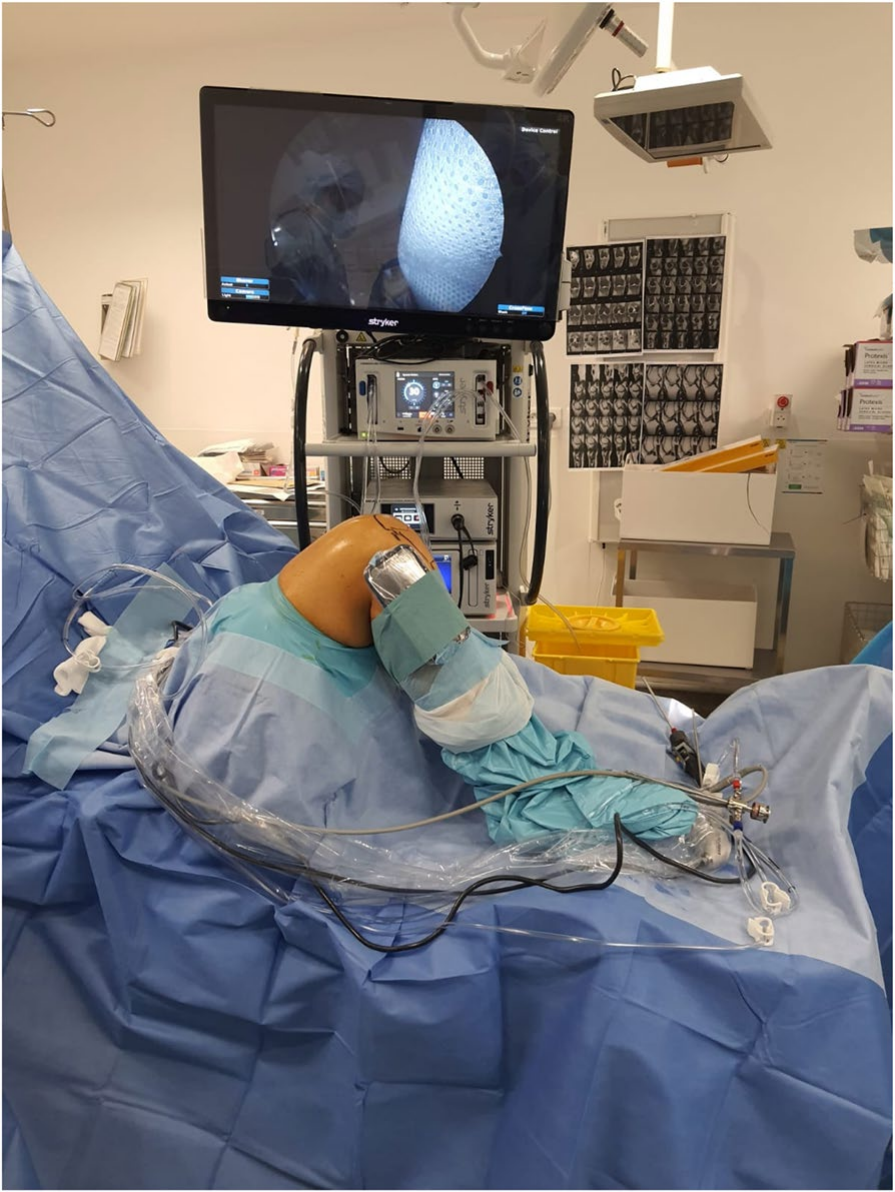
The smartphone was placed in a sterile bag and sealed airtight to maintain sterility and placed on the Gerdy’s tubercule

As per the CLIN (committee for the control of nosocomial infections)protocol which is defined by the institution, the injured limb was prepared utilizing the standard antiseptic techniques. Next, the smartphone was placed in a sterile bag and sealed airtight to maintain sterility (Fig. 1).

The PS test procedure was then repeated, three times during each per-operative stage (ACL reconstruction, and following LET or meniscal repair), and post-operatively, by the same two orthopedic surgeons. The PS test was also evaluated clinically according to the IKDC classification.

The smartphone (Pro Mate 20, Huawei, Shenzhen, China) with the Sensor Kinetics Pro application (INNOVENTIONS Inc., Houston, TX, USA) was attached to Gerdy’s tubercle with adhesive strips. The Y axis of the phone was parallel to the axis of the tibia. This protocol, already detailed in a previous study [29], allows the analysis of anteroposterior translation and acceleration during the reduction of the lateral compartment of the knee in the PS test.

**Fig. 2:**
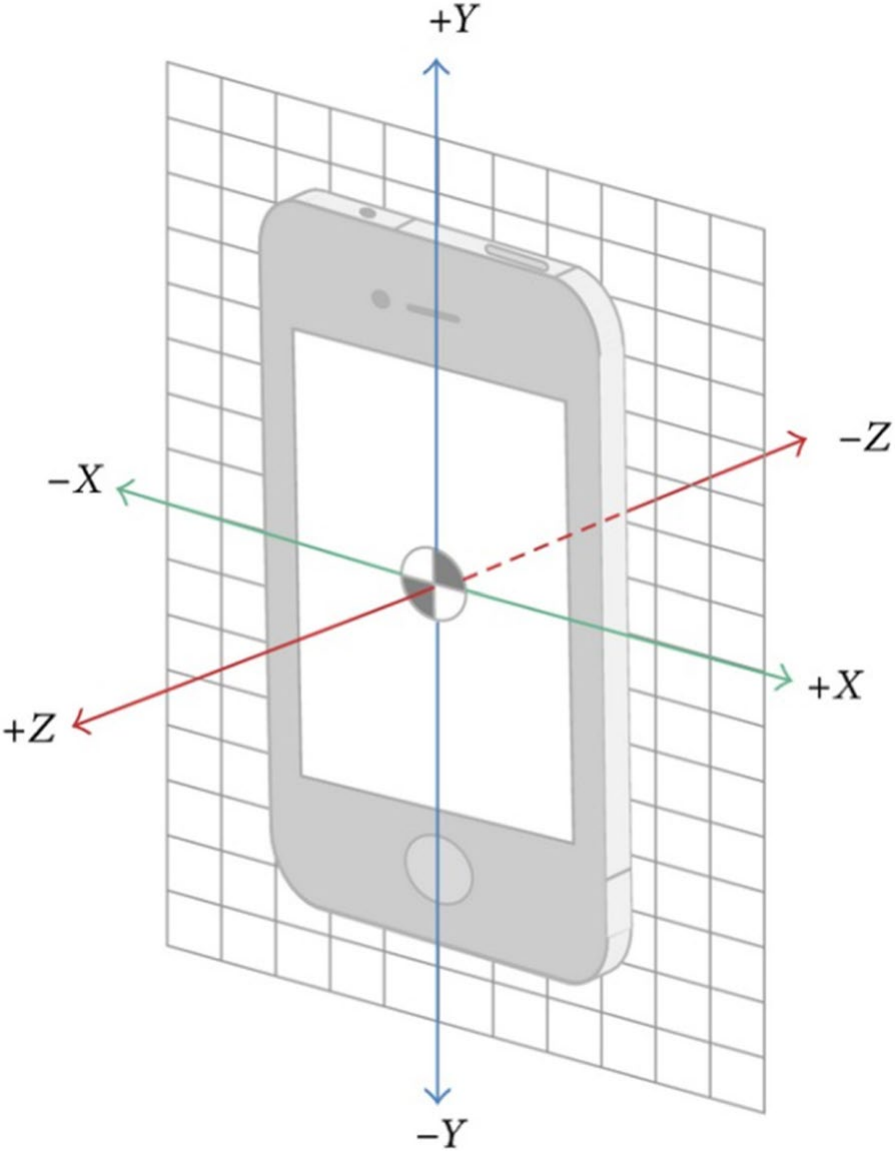
Representation of the different axes of the smartphone accelerometer in space

**Fig. 3:**
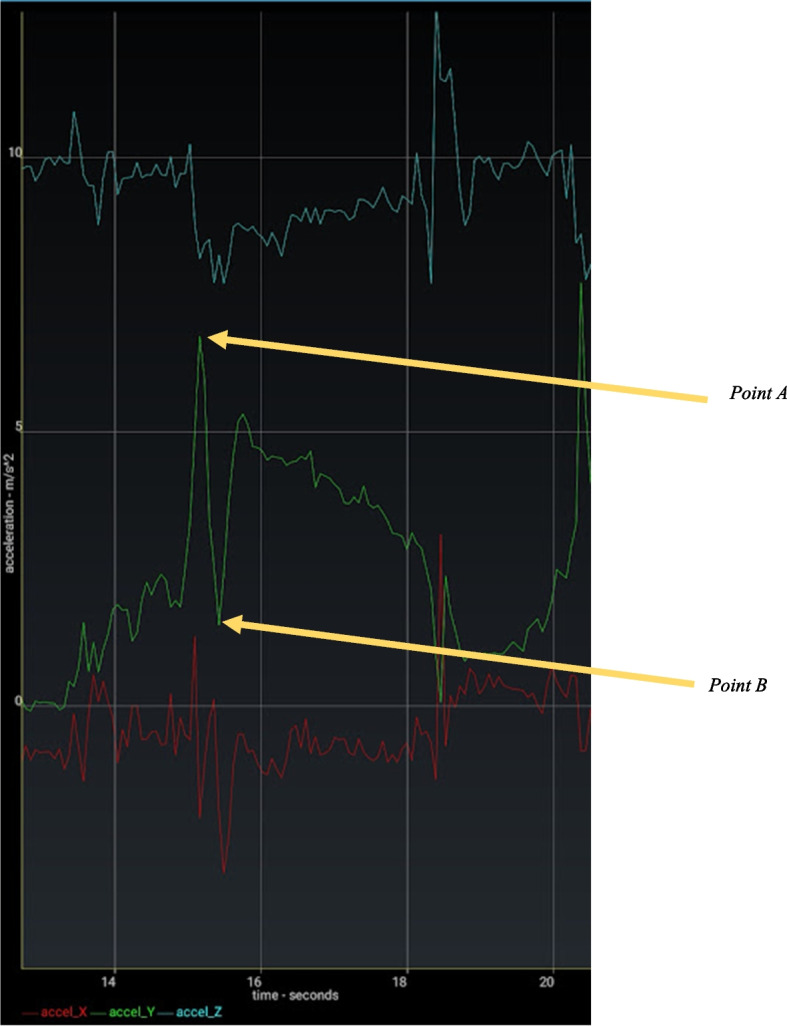
Evolution of acceleration during the PS test on a pathological knee with an anterior cruciate rupture. The Y axis (green) showed the flexion-extension movement induced by the maneuver. The subluxation and abrupt reduction of the anterior compartment of the tibia was highlighted by the abrupt deceleration with point A corresponding to the maximum of the subluxation and point B the reduction. The difference between point A and point B was called the alpha value

The Sensor Kinetics Pro application was downloaded to the smartphone. The application measured the acceleration of the movement of the tibia relative to the femur along the X, Y and Z axes of the accelerometer quantitatively in m.s^2^ (Fig. 2) In this study, the Y-axis represented the acceleration of the longitudinal axis of the tibia, the X-axis the acceleration in the anteroposterior plane relative to the longitudinal axis, and the Z-axis the acceleration in the mediolateral plane. An evolving graph of the 3 axes was displayed directly on the screen during all the acquisition and the different manipulations (Fig. 3). The measurements were recorded in an .acc graphics files and renamed by a number to guarantee the anonymity of the patient. This data allowed us to analyze the changes, induced by the surgical procedure, on the knee kinetics and instability.

### Statistical analysis

According to Kocher et al. [11], a change in acceleration of 1.6 m.s^2^ was significant (ruptured ACL 4.3 +/− 1.2 m.s^2^, healthy knee 2.7 +/− 0.7 m.s^2^). According to the original study [29], the Y-axis showed statistically the best inter- and intra-observer reproducibility, and according to the authors, best represented the direction of tibial acceleration during the PS.

The analyses were performed using Python (3.10.4) from the Company software. Intra- and inter-observer reliability was evaluated via intra-class correlation coefficient (ICC) analysis, in absolute agreement two-way mixed-effect model with single fixed rates. Reliability was defined as poor (ICC < 0.5), moderate (0.5 < ICC < 0.75), good (0.75 < ICC < 0.90) and excellent (ICC < 0.90). The standard error of measurements (SEM) were also reported. Abnormal distribution of continuous variables was verified using the Shapiro Wilk test. Data was expressed as means ± standard deviation (SD). The Spearman correlation was used to correlate the anterior tibial translation with the IKDC ranking grade. One-way analysis of variance was performed to determine whether the range of acceleration was significantly different among the clinical grading categories (0, 1, 2, or 3).

## Results

After clinical examination of 12 knees were finally included in this study,

The mean measurements (of both examiners) of PS test during the different steps of the surgery are summarized in the Table 1. The intra-observer reliability was good for the first examiner and excellent for the second examiner, with the ICC 0.89 [0.67, 0.98] *p* < 0,001 and ICC 0.97 [0.91, 1.0] *p* < 0,001 respectively.

**Table 1:** Average measurements (of both examiners) and standard deviation of pivot shift during the different stages of intervention

Mean measurement of the PS (m.s^2^)						
Contralateral pivot shift	Preoperative pathological pivot shift	Pivot shift after ramp repair	Pivot shift after internal meniscal injury repair	Pivot shift after external meniscal injury repair	Pivot shift after ACL reconstruction	Pivot shift after lateral tenodesis
0.8 (0.1)	2.6 (0.37)				1.4 (0.56)	0.9 (0.22)
1.1 (0.39)	2.9 (0.53)		2.1 (0.21)		1.8 (0.31)	1.1 (0.17)
0.8 (0.24)	0.8 (0.19)		0.9 (0.22)		0.7 (0.14)	0.7 (0.16)
0.7 (0.21)	3.6 (0.3)			3.4 (0.36)	2.5 (0.29)	1.1 (0.49)
0.9 (0.14)	1 (0.38)				0.9 (0.18)	0.9 (0.29)
1.4 (0.31)	5 (0.48)	3.2 (0.57)			2.3 (0.33)	1.4 (0.37)
0.9 (0.18)	1.2 (0.43)		1.2 (0.27)	1 (0.24)	1 (0.22)	1.1 (0.17)
0.6 (0.29)	4.4 (0.4)			2.2 (0.15)	1.4 (0.47)	1.1 (0.4)
0.9 (0.29)	4.3 (0.77)			2.5 (0.33)	2.4 (0.18)	1 (0.21)
0.9 (0.21)	5.5 (0.25)			2.3 (0.17)	1.2 (0.17)	0.8 (0.18)
0.7 (0.18)	4.5 (0.23)			2.8 (0.17)	2.1 (0.15)	1.1 (0.12)
0.8 (0.18)	5.5 (0.7)			3.8 (0.3)	1.6 (0.1)	0.9 (0.15)

The inter-observer reliability was excellent between the two observers with the ICC 0.99 [0.97, 1.0] *p* < 0,001.

The mean value of the tibial acceleration during PS test for the ACL-injured knees was 3.45 m.s2 (SD = 1.71), which was significantly higher than that of normal knees (1.03 m.s2; SD = 0.36, *p* < 0.001).

At the end of the 12 interventions performed, all PS tests were graded 0 in accordance with the IKDC classification.

**Fig. 4:**
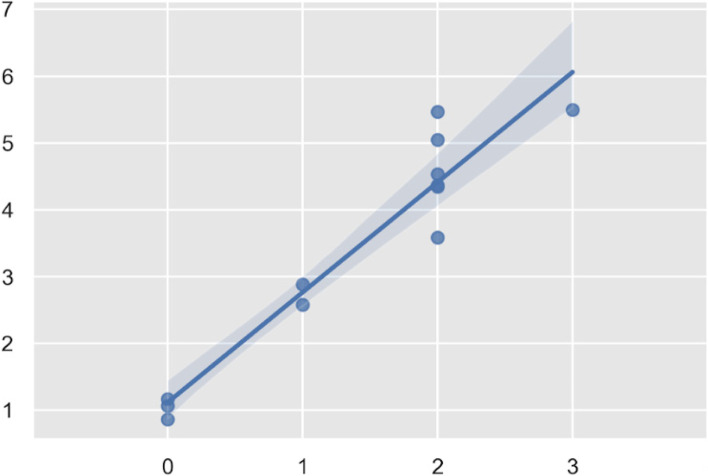
Forest plot of the mean values of tibial acceleration in Y axis according to the IKDC grade of PS in X axis

The correlation between the IKDC grade of PS and the range of value of tibial acceleration was 0,927, p 10^− 5^. A forest plot was shown in Fig. 4.

## Discussion

The main finding of the present study is that the use of a smartphone accelerometer intra-operatively to quantitatively characterize the subjective PS during ACL reconstruction surgery is validated with excellent interclass correlation coefficients between the two surgeons and good or excellent intraclass correlation. The results of the present study showed an evolution of the quantitative assessment of the PS test performed intra-operatively. The results presented in this study also considered the involvement of the meniscoligamentous injuries in the magnitude of the PS test. No single stage of the procedure demonstrated a significant reduction in the acceleration of the tibia on the femur between meniscal repair, ACL reconstruction, ramp repair and lateral tenodesis. We assumed that the population size was too small to show a statistical significance and that a larger trial would be required to answer this question.

Numerous studies have looked at the relationship between PS and the patient’s meniscal status. The study by Jacquet et al. [10] presented risk factors for PS during clinical follow-up and highlighted the protective effect of meniscal repair, the deleterious effect of meniscectomy, therapeutic abstention or the appearance of a new meniscal lesion. Moreover, the presence of a high-grade PS was a predictive factor for the development of new meniscal injuries. The study by Mouton et al. [18] showed a greater PS in patients with a ramp lesion. The relationship between lateral tenodesis and PS has been more controversial in the literature. The ACL Consensus group [6] recommended that a high-grade preoperative PS could be an indication for an additional anterolateral procedure in the setting of ACL reconstruction. In contrast, Jacquet et al . [10] found that the addition of a lateral tenodesis at the time of the operation did not influence PS. However, in a recent study, Firth et al . [4] showed that the addition of LET reduces the odds of postoperative asymmetric PS by 46%. The results of this study showed that meniscal repairs, especially ramp and external meniscal injury repairs, decreased the PS acceleration and in most cases, lateral tenodesis had a little impact, even though the results were not significant.

This study had several limitations. First of all, the PS test is dependent on the surgeon’s experience, and grading it according to the IKDC classification is subjective. Berruto et al. [2] reported a learning curve, when measuring the tibia acceleration (a 50% specificity at the beginning to 90% specificity at the end of the study) attributed to their device (KiRA), but it could be attributed to the performance of the PS test [20]. Therefore, in this present study, the whole technique was well documented and described [21]. The same technique was taught and performed [8, 19] in order to limit this bias, but could not provide a total emancipation.

Another potential limitation may be related to the choice of the studied parameter. The acceleration of the longitudinal translation of the tibia during the maneuver was considered the key to quantify the extent of the PS, whereas the smartphone’s application could measure multiple other parameters which may contribute to the PS. The acceleration of the longitudinal translation of the tibia seemed to be the most reliable [29], whereas the rotational component lacked precision. Indeed, Vaidya et al. showed an AUC value of 0,98 and a specificity of 100% for the Y-axis with a smartphone accelerometer, better than other commercial accelerometers [2, 8, 22], and similar to electromagnetic devices [27]. A cadaveric study highlighted that the anterior tibial lateral compartment translation of 6-7 mm is necessary for a grade 1 PS, 15 mm for a grade 2, and over 20–25 mm for a grade 3 [1]. The use of this parameter is supported with strong evidence in literature [2, 7, 22, 29]. Berruto et al. [2] found a positive acceleration difference in favor of the pathologic knee among 100 patients and a reliable use of KiRA, another triaxial accelerometer, with mean reference values for every grade of the PS test.

A recent study [22] did not support the use of the triaxial accelerometer alone to assign a numerical value to the pivot-shift phenomenon. At first, parameters other than acceleration range were evaluated and appeared unable to show correlation with clinical examination. Moreover, the direction of the acceleration range was unspecified, although Vaidya et al. [29] showed statistical difference between the longitudinal (Y axis) and the others. However, the PS is still described as a translation corresponding to anterior subluxation of the lateral tibial plateau, which was measured, and a rotation of the tibia relative to the femur [15] which is fully occulted in biomechanical studies. Further studies should be conducted to use the included gyrometer in order to better understand the rotational laxity.

This study also presents some strengths. At first, to the best of our knowledge, no other study provided an intraoperative quantitative assessment of the PS. Most of the devices on the market are either invasive, bulky or expensive, such as electromagnetic devices [13]. The most commonly used and studied system, the KiRA, cannot be used intraoperatively. Smartphones provided a cheaper solution within everyone’s reach. A protocol is proposed to maintain sterility for it use intraoperatively. The use of this tool could help in the decision to perform a tenodesis if the measured rotational instability is not fully corrected by meniscal repairs and ACLR.

## Conclusion

The smartphone accelerometer is a reproducible device to quantitatively assess the internal rotation and anterior tibial translation during ACL reconstruction surgery. The measurements are influenced by the different surgical steps. Other larger cohort studies are needed to evaluate the specific impact of each step of the ACL reconstruction and meniscal repair on this measurement. An external validation using other technologies are needed to validate the reliability of this device to assess the PS test.

## Data Availability

Yes
